# The Artificial Illumination of the Small Hospital

**Published:** 1911-05-20

**Authors:** 


### THE ARTIFICIAL ILLUMINATION OF THE SMALL HOSPITAL.

### SOME COMPARATIVE POINTS ON PETROL, GAS, AND ACETYLENE.

In the erection of new buildings, and sometimes of existing buildings, the question arises as to whether the means of illumination will be taken from the town mains (gas or electric) or whether it will prove more economical to instal a generating plant.

For a building situated where there is no town supply convenient, and where the number of lights is small, probably the modern incandescent petrol-lamp will answer the purpose perfectly. Its consumption costs much less than the old-fashioned paraffin lamp, as will be readily understood from the fact that 98½ parts of air go to 1½ part of vapour consumed, and with this small consumption a 60-candle-power light is given. This type of lamp has no wick to be trimmed, a constant source of worry with a paraffin lamp, and, further, there is no danger of explosion, as this lamp, when it is upset, goes out. A pattern called "Petrolito" was submitted to the British Fire Prevention Committee and in the test was dropped among muslin, shavings, and other highly inflammable materials, but the lamp simply went out, and every possible means of creating a fire or mishap failed; its excellence also gained its patentees an award from the Royal Society. The cost of a lamp as described is from 12s. 6d. upwards, depending on the amount of elaboration required in the ornamental fittings.

If coal gas is available, and it is decided to use this as an illuminant, the question will arise as to what type of burner gives the best results. Ordinary burners show a consumption of about 4 cubic feet per hour; the incandescent burner uses up not less than 2½ cubic feet per hour, whereas the inverted incandescent burner consumes only $1\frac{1}{10}$ cubic foot.

It must be remembered in fixing brackets or standards that the burner should not be within 3 feet of a ceiling or nearer to any woodwork than 10 inches. The quality and class of the burner is of the utmost importance if good illumination is to be the result and a bad light may generally be put down to a defective burner rather than to inferior quality of gas.

The incandescent burner is a modified and improved Bunsen burner, the flame of which is enclosed within a mantle formed of a mixture of rare earths; it contains 1 to 2 per cent. of cerium, the remainder being an oxide of thorium. It is owing to the great heat evolved by the perfect combustion of the gas that the surface of the mantle is raised to a state of incandescence so brilliant that with a consumption of 5 feet per hour a light equal to 150 candles can be obtained. As the efficiency of a Bunsen burner largely depends upon the amount of air sucked in by the jet of gas, it is of interest to compare the volumes respectively drawn in by a Bunsen burner, the Welsbach C burner, and the new Welsbach burner.

| Description of Burner. | Volume of Gas. | Volume of Air. |
|---|---|---|
| 1. Bunsen burner just non-luminous ... ... | 1 | 2.3 |
| 2. Welsbach C burner ... ... ... ... | 1 | 3.0 |
| 3. Welsbach new burner ... ... ... ... | 1 | 4.5 |

Recently there has appeared on the market a type of burner known as the Hella bushlight, which, it is claimed, is as great an advance over the ordinary incandescent mantle as the latter was over the old-fashioned bat's-wing burner. One if its salient advantages is the strength of the mantle, which will stand wind, rain, or vibrations without breaking. This mantle is obtained by mixing certain rare earths and forming them into a plastic mass, which is submitted to enormous pressure, and filaments of thickness varying from $\frac{2}{10}$ mm. to $1\frac{1}{2}$ mm. are produced. These filaments are subjected to a very high temperature, when the constituents fuse and become inseparable and imperishable. Every particle of these fused earths has the property of becoming incandescent when submitted to heat, and this should tend to a constant radiance, or, in other words, to an economy of gas by the more perfect combustion. The cost of this burner, complete, is half-a-crown.

Owing to the richness of acetylene gas in carbon it requires the presence of a larger volume of air to ensure complete combustion; if the air-supply is deficient the burners become carbonised and clogged, the flame is smoky, and pure lamp-black is given off. To obviate this defect fine holes are drilled radially just below the top of the burner, and the stream of gas sucks in sufficient air on its way to the orifice. Ordinary metal burners proving unsatisfactory, experiments were made with other materials; that now mostly employed is called "steatite," and the burners are made of it wholly or in part.

The best form of burner is the double-arm, or horseshoe type, in which small streams of gas from either jet impinge on each other; the flame does not touch the burner, but is suspended, as it were, between the two arms.

The following table gives approximate costs of lighting by different methods:—

| | |
|---|---|
| Coal gas (ordinary burner) at 2s. 9d. per 1,000 cubic feet... | 1.00 |
| Coal gas (bat's-wing burner) at 4s. per 1,000 cubic feet ... | 1.45 |
| Welsbach incandescent (exclusive of mantles) ... ... | 0.98 |
| Acetylene (carbide at £20 per ton) ... ... ... ... ... | 0.85 |
| Petroleum ... ... ... ... ... ... ... ... ... | 0.62 |

